# Ten Simple Rules for Chairing a Scientific Session

**DOI:** 10.1371/journal.pcbi.1000517

**Published:** 2009-09-25

**Authors:** Alex Bateman, Philip E. Bourne

**Affiliations:** 1 Wellcome Trust Sanger Institute, Cambridge, United Kingdom; 2 Skaggs School of Pharmacy and Pharmaceutical Science, University of California San Diego, La Jolla, California, United States of America

Chairing a session at a scientific conference is a thankless task. If you get it right, no one is likely to notice. But there are many ways to get it wrong and a little preparation goes a long way to making the session a success. Here are a few pointers that we have picked up over the years.

## Rule 1: Don't Let Things Overrun

Probably the main role of the session chair is to keep the meeting running on time. Time is a strange and elastic concept when people are under pressure. Some speakers will talk much faster than normal and finish a talk in half the expected time. Others will ramble on without knowing that time is running out and they have only just finished their introduction. Timing is important to ensure that a meeting runs smoothly. Delegates should leave the session at just the right time so that lunches are still fresh, bars still open, etc. Timing is particularly acute if there are multiple parallel sessions and delegates would want to switch between talks in different sessions.

## Rule 2: Let Your Speakers Know the Rules

A session will run more smoothly if you let all the speakers know how you plan to run your session. This could be done by e-mail before the event or you might want to gather up the speakers just before the session. Reminding them how much time they have to speak, how much time to allow for questions, and how you will let them know time is up will stop confusion later on. Beyond the rules, encourage speakers to review what others in the session will say. The less redundancy, the better the session will be for everyone, including the chair.

## Rule 3: Be Prepared to Give a Short Introduction

Be prepared to give a short introduction to the session, and, of course, introduce yourself as well. Be sure to review the abstracts of the talks and then give a succinct summary of what will be presented. It is your job to excite people at the session and have them stay in the auditorium. Regarding the speakers, introduce each one before they begin, providing their background and highlighting their major accomplishments. Speakers love to be properly introduced and the audience likes to feel they know the person speaking. But for the sake of both the timing of the session and your speakers, do keep it brief. Are you expected to give any housekeeping messages or to remind people to switch off their phones? Allow time for that if so.

## Rule 4: Write Down the Actual Start Times of the Speakers

If you don't know what time a speaker started, it is difficult to know when to ask them to stop. So always write down the start and finish times of speakers throughout the session.

## Rule 5: Do Have a Watch

It sounds obvious, but it is very difficult to chair a session if you don't have a watch and don't know the time. Yes, one of us has done this! It is embarrassing to have to ask your neighbor for a watch. Actually, it is probably best to have two watches, just in case.

## Rule 6: Communicate How Much Time is Left to the Speaker

Letting the speaker know their time is up is crucial in keeping time. A simple sign held up at the right time is usually fine. Have one saying, “5 minutes to go” and another saying “time is up”. Beyond that time, standing up on the stage is a good sign that the speaker should wrap up.

## Rule 7: Don't Be Afraid to Move on Without Questions

A good scientific session is characterized by a lively question and answer session. In fact, some speakers believe it is their right to expect to answer questions even after their allotted time is up. If you are running over time, you should not be afraid to move on to the next talk without questions. You will be more confident in enforcing this principle if you have warned the speaker beforehand that running over will require foregoing taking questions at that time. You can stay on schedule by diplomatically saying that the speaker will be happy to take questions at the break.

## Rule 8: Get to the Venue Early and Be Audiovisually Aware

Make sure to know where everything is, like pointers, microphones, projectors, and computers and who to turn to if it all goes wrong. It is worth checking that all these things work so that you can swiftly fix them yourself. Knowing ahead of time any unusual requests from speakers to show movies and sound clips requiring special attention. Be sure the venue supports the needs of speakers. If not, let them know before they get to the venue. If each speaker is expected to load their presentation on a single computer associated with the podium, allow time for that and have the speaker run through their slides to be sure everything is working properly.

## Rule 9: Prepare Some Questions in Advance

It can take an audience a few seconds to digest the contents of a talk and think of questions. So, it is always good to have one or two ready to ask. These can be prepared beforehand from the abstracts and supplemented from ones that occur to you during the talk. This is a very good reason for paying attention during the talk. Also, it is worth thinking of one or two general purpose questions such as “What do you plan to do next?”

## Rule 10: Keep Control of the Question and Answer Sessions

It is difficult for the session chair to keep things on time if the speaker is in control of taking questions. Make sure you are the one who selects the next questioner. Also, be prepared to step in if the speaker and questioner are getting into a long-winded, technical discussion.

Hopefully with a bit of preparation and a little luck, you will get through the ordeal of chairing a scientific session unscathed. And remember, if no one thanks you, you have probably done an excellent job.

